# Embryonic Lethality Due to Arrested Cardiac Development in *Psip1/Hdgfrp2* Double-Deficient Mice

**DOI:** 10.1371/journal.pone.0137797

**Published:** 2015-09-14

**Authors:** Hao Wang, Ming-Chieh Shun, Amy K. Dickson, Alan N. Engelman

**Affiliations:** Department of Cancer Immunology and AIDS, Dana-Farber Cancer Institute and Department of Medicine, Harvard Medical School, Boston, Massachusetts, 02215, United States of America; Nanjing Medical University, CHINA

## Abstract

Hepatoma-derived growth factor (HDGF) related protein 2 (HRP2) and lens epithelium-derived growth factor (LEDGF)/p75 are closely related members of the HRP2 protein family. LEDGF/p75 has been implicated in numerous human pathologies including cancer, autoimmunity, and infectious disease. Knockout of the *Psip1* gene, which encodes for LEDGF/p75 and the shorter LEDGF/p52 isoform, was previously shown to cause perinatal lethality in mice. The function of HRP2 was by contrast largely unknown. To learn about the role of HRP2 in development, we knocked out the *Hdgfrp2* gene, which encodes for HRP2, in both normal and *Psip1* knockout mice. *Hdgfrp2* knockout mice developed normally and were fertile. By contrast, the double deficient mice died at approximate embryonic day (E) 13.5. Histological examination revealed ventricular septal defect (VSD) associated with E14.5 double knockout embryos. To investigate the underlying molecular mechanism(s), RNA recovered from ventricular tissue was subjected to RNA-sequencing on the Illumina platform. Bioinformatic analysis revealed several genes and biological pathways that were significantly deregulated by the *Psip1* knockout and/or *Psip1*/*Hdgfrp2* double knockout. Among the dozen genes known to encode for LEDGF/p75 binding factors, only the expression of *Nova1*, which encodes an RNA splicing factor, was significantly deregulated by the knockouts. However the expression of other RNA splicing factors, including the LEDGF/p52-interacting protein ASF/SF2, was not significantly altered, indicating that deregulation of global RNA splicing was not a driving factor in the pathology of the VSD. Tumor growth factor (Tgf) β**-**signaling, which plays a key role in cardiac morphogenesis during development, was the only pathway significantly deregulated by the double knockout as compared to control and *Psip1* knockout samples. We accordingly speculate that deregulated Tgf-β signaling was a contributing factor to the VSD and prenatal lethality of *Psip1*/*Hdgfrp2* double-deficient mice.

## Introduction

Members of the hepatoma-derived growth factor (HDGF) related protein (HRP) family have been implicated in numerous human diseases including cancer, autoimmunity, and virus infection [1, 2]. The family consists of six members including HDGF, HRP1, HRP2, HRP3, and lens epithelium-derived growth factor (LEDGF)/p52 and LEDGF/p75, the latter two of which are expressed from the PC4 and SFRS1-interacting protein 1 (*Psip1*) gene as splice variants [3]. The most common feature among these proteins is an N-terminally situated PWWP (Pro-Trp-Trp-Pro) domain [4], which is a type of Tudor domain [5]. Two of the proteins, LEDGF/p75 and HRP2, contain a second evolutionarily conserved domain, which we previously termed the integrase-binding domain because it was both necessary and sufficient to convey binding to lentiviral integrase proteins [3, 6, 7]. LEDGF/p75, which is an important lentiviral integrase cofactor, helps to guide the integration of the viral reverse transcripts into the bodies of active genes (see [1] and [8] for review).

Despite possessing a functional integrase-binding domain [3], HRP2 unlike LEDGF/p75 does not appear under normal circumstances to play a role in lentiviral DNA integration. A subsidiary role for HRP2 in guiding human immunodeficiency virus type 1 (HIV-1) integration to active genes was however uncovered by either knocking down [9] or knocking out [10] the expression of HRP2 in *Psip1* knockout cells that are completely devoid of LEDGF/p75 expression. Two differences between HRP2 and LEDGF/p75 are likely to contribute to the inability for HRP2 to contribute to viral DNA integration under normal circumstances. Firstly, whereas LEDGF/p75 associates with chromatin throughout the cell cycle [11–13], HRP2, though localized to the cell nucleus, does not associate with chromatin [13, 14]. Secondly, the affinity of the HRP2-integrase interaction is lower than the affinity of the LEDGF/p75-integrase interaction [3, 13].

We previously knocked out *Psip1* [15] and *Hdgfrp2* (the gene that encodes for HRP2) [10] in mice to generate mouse embryo fibroblasts (MEFs) as cell models for HIV-1 infection. In this report we detail the phenotypes of the knockout animals. Bickmore and colleagues previously described that the majority of *Psip1* knockout mice generated via gene trap vector insertion died perinatally [16], a result recapitulated here through Cre/lox-mediated disruption of the *Psip1* gene. We additionally found that *Psip1*/*Hdgfrp2* double deficient mice displayed a high rate of prenatal mortality, with the underlying pathology of ventricular septal defect (VSD) mapping to approximate embryonic day (E) 13.5. Illumina-based sequencing of RNA transcripts (RNA-Seq) derived from tissues of *Psip1* knockout, *Psip1*/*Hdgfrp2* double knockout, and littermate-matched control animals revealed genes and pathways that were deregulated by the double knockout including tumor growth factor (Tgf)-β signaling, extracellular matrix (Ecm)-receptor interaction, and focal adhesion. Deregulation of these pathways may contribute to the VSD and the mortality of the mice.

## Material and Methods

### Knockout animals and MEF cells

The procedures used to generate *Psip1*, *Hdgfrp2*, and *Psip1*/*Hdgfrp2* knockout mice were described previously [10, 15]. In brief, LEDGF/p75 expression was knocked out by deleting exon 3 of the *Psip1* gene, which is the second coding exon, using Cre/lox-mediated DNA recombination [15]. Embryonic stem (ES) cells that harbored the insertion of the pGT2lfx gene trap vector between exons 3 and 4 of *Hdgfrp2* were obtained from BayGenomics [10]. Chimeric animals obtained from implanting ES cells that were disrupted for either the *Psip1* or *Hdgfrp2* gene were backcrossed to C57BL/6 mice (Charles River Laboratories) to yield heterozygous *Psip1* (+/-) and *Hdgfrp2* (+/g) animals. The heterozygous mice were mated to yield *Psip1* (-/-) knockout, *Hdgfrp2* (g/g) knockout, or heterozygous +-/g+ and +-/gg animals, the latter of which were further interbred to yield −−/gg double knockout animals [10]. The statistical relevance of observed frequencies of mouse genotypes was assessed versus the expected Mendelian frequencies using the chi-square test.

The E9 and E13 sets of WT (++/+g), *Psip1* knockout (−−/+g), and *Psip1*/*Hdgfrp2* double knockout (−−/gg) MEF cell lines were previously described [10].

### PCR analysis of mouse genomic DNA

Genotyping was performed by Southern blotting and by PCR [10, 15]. In brief, DNA prepared from mouse tissue using the QIAamp DNA micro kit (Qiagen) was PCR-amplified using primers AE2331 and AE2802 to monitor the status of the *Psip1* gene [15] and primer pairs AE2511/AE2512 and AE3747/AE3748 to monitor the *Hdgfrp2* locus [10]. S1 Table lists the sequences of the PCR primers that were used in this study.

The sex-determining region Y (*Sry*) gene was amplified using primers AE6796/AE6797 and 50 ng genomic DNA under cycling conditions: 98°C for 5 min, followed by 30 cycles of 30 sec at 98°C, 30 sec at 56°C, 15 sec at 72°C, followed by a final 10-min extension at 72°C. The resulting 273 bp *Sry*-specific amplification product was visualized by staining with ethidium bromide following agarose gel electrophoresis.

### Quantitative (q) RT-PCR

The concentration of RNA extracted from mouse tissue using the RNeasy Mini Kit (Qiagen) was determined by spectrophotometry. Duplicate qRT-PCR mixtures contained 0.5 μM primers, 1×Quantitect SYBR green master mix, 0.3 μl QuantiTect RT mix (QuantiTect Sybr Green RT-PCR kit), and 25 ng of RNA. *Psip1* expression was monitored using primers AE2624/AE2625, which anneal to exons 2 and 3 [15]. Primers AE3160/AE3161 were used to amplify *Hdgfrp2* exons 1/2 whereas downstream exon 5/7 sequences were amplified using primers AE2553/2554 [10]. Gene expression data were normalized to *Ppia*, which encodes for cyclophilin A, using primers AE3664/AE3665 [10]. PCR cycling conditions were as described [10]. Significance between levels of gene expression was assessed by one-tailed t test.

### RNA-Seq analysis

Embryo hearts were dissected from euthanized E14.5 animals, and the ventricular tissue was isolated from the atrial chambers. RNA extracted from the ventricles using the RNeasy kit was subjected to the RNA-Seq pipeline at the Center for Cancer Computational Biology (Dana-Farber Cancer Institute). RNA was analyzed for quality control using Qubit fluorometric quantitation (Life Technologies) and Bioanalyzer (Agilent Technologies). RNA (50–100 ng) was converted into DNA libraries using the NEBNext Ultra RNA Library Prep Kit for Illumina (New England Biolabs). The quality of the DNA libraries was assessed using the Qubit High Sensitivity DNA Kit (Life Technologies) and library size was determined using the Bioanalyzer High Sensitivity Chip Kit (Agilent Technologies). Libraries that passed quality control were diluted to 2 nM using sterile water and then sequenced on the MiSeq2000 platform (Illumina) at the concentration of 12 pM on a single read flowcell with 50 sequencing cycles.

### Western blotting

Mouse tissue was lysed in RIPA buffer (50 mM Tris-HCl, pH 7.4, 150 mM NaCl, 1% Triton X-100, 1% sodium deoxycholate, 0.1% sodium dodecyl sulfate, 1 mM EDTA) containing 1x protease inhibitor cocktail (Roche Diagnostics). The samples were subjected to sonication for 5 to 10 sec, followed by centrifugation at 16,000 g for 5 min at 4°C. The concentration of supernatant protein was determined using the Bio-Rad DC Protein Assay Kit, and 5 μg was fractionated through 8% polyacrylamide gels under denaturing conditions. Proteins transferred to poly(vinylidene difluoride) membrane were probed with anti-Smad2/3 antibodies (Cell Signaling) at 1:1,000 dilution. Primary antibody binding was visualized using horseradish peroxidase-conjugated rabbit anti-mouse antibodies (Dako Scientific) and enhanced chemiluminescence (Thermo Scientific). Membranes were reprobed with horseradish peroxidase-conjugated antibody against β-actin (1:10,000 dilution, Cell Signaling) to control for the amount of protein loaded onto the gels. Membranes were imaged on a ChemiDoc MP imager (Bio-Rad) and signals were quantified using Image Lab 4.1 software.

### Bioinformatics and statistical analyses

Sequence reads were mapped to *Mus musculus* reference genome mm9 (build name NCBIM37) using TopHat [17]. HTSeq was used to map the reads to each gene from the alignment BAM file [18]. EdgeR was used to analyze differential gene expression [19]. Read counts from triplicate RNA samples were first normalized to library size, then relatively low expressed genes were filtered out by using the threshold setting of one read per kb per million reads (RPKM). Testing for differentially expressed genes was based on a negative binomial model. Significant differential expression was filtered using a false discovery rate of < 0.05. The top 20 differentially expressed genes were additionally sorted by *P* value.

To analyze gene ontology, the differentially expressed genes identified using EdgeR were processed by the online tool provided by www.biomart.com. Gene set and pathway analysis was performed using the Generally Applicable Gene-set Enrichment (GAGE) [20] package and the results were presented in KEGG pathway [21, 22]. Significantly regulated pathways were filtered using a q value of < 0.1. Pathview [23] was utilized to visualize results as indicated.

### Histological analysis of mouse tissue

Mouse tissue was fixed in Bouin’s fixative (Sigma-Aldrich). Tissues were embedded in paraffin, sectioned at 6 μm, stained with hematoxylin/eosin, and analyzed by light microscopy at the Rodent Histopathology Core at Harvard Medical School.

### Ethics statement

This study was carried out under strict supervision of the Beth Israel Deaconess Medical Center (BIDMC) Institutional Animal Care and Use Committee (IACUC) under guidelines set forth by the United States Department of Agriculture (USDA) and US Public Health Service (PHS) Office of Lab Animal Welfare (OLAW). The BIDMC IACUC, which is accredited by the Association for Assessment and Accreditation of Laboratory Animal Care (approval date March 25, 2014) and US PHS Assurance code A3153-01 (expiration date February 28, 2018), approved this study as part of animal protocol number 038–2012 (“Breeding LEDGF and HRP2 knockout mice”).

Mice were identified using ear punches and the universal mouse numbering system in accordance with Institutional and Federal guidelines. Tips (2–4 mm) of mice tails were snipped for genotyping purposes using an extremely sharp blade (scalpel or razor blade), and hemostasis was performed before returning the animals to cages to minimize any associated discomfort. Pregnant mothers were euthanized by CO_2_ inhalation for 10 min using a smart box chamber system (Euthanex).

## Results and Discussion

### Generation of knockout mice

Several strains of mutant mice were generated to investigate the roles of *Psip1* and *Hdgfrp2* in mouse development. *Psip1* was knocked out using Cre/lox DNA recombination [15] and *Hdgfrp2* was disrupted by gene trap insertion [10]. Heterozygous animals (+/- for *Psip1*; +/g for *Hdgfrp2*) were then interbred to generate *Psip1*/*Hdgfrp2* double knockout animals. Because *Hdgfrp2* (g/g) knockout animals reached adulthood (see below) and were fertile, +-/+g heterozygous animals were mated to +-/gg *Hdgfrp2* knockout animals to increase the theoretical frequency of double knockout production from 6.25% to 12.5% of offspring. All animals generated from the double knockout mating scheme were therefore either *Hdgfrp2* (+/g) or *Hdgfrp2* (g/g).

The genotypes of knockout animals were monitored using PCR and Southern blotting whereas gene expression profiles were monitored by qRT-PCR and western blotting [10, 15]. Examples of these measurements for animals generated by the double knockout mating scheme are presented in S1A–S1C Fig. Two sets of qRT-PCR primers were used to monitor *Hdgfrp2* expression: exon 1/2-specific primers detected sequences upstream from the gene trap insertion, whereas exon 5/7 primers monitored expression downstream from the insertion. The gene trap vector reduced the expression level of exon 1/2-containing sequences approximately 5 fold relative to a ++/+g littermate-matched control animal, whereas exon 5/7-contaning sequences were further reduced, to about 15-to-25 fold, compared to the control. The level of *Hdgfrp2* expression was by contrast unaffected by *Psip1* knockout in the +/g background (S1B Fig). *Psip1* knockout reduced the level of LEDGF/p75 mRNA between 300 and 1000 fold, whereas *Psip1* heterozygosity (+/-) yielded an approximate 2-fold reduction in message (S1C Fig). The strength of gene trap knockout depends on the position of vector integration in the mouse genome [24, 25] and we accordingly expect that this parameter impacted the magnitude of the *Hdgfrp2* mRNA reduction as compared to the more potent reduction in *Psip1* message that was achieved through Cre/lox-mediated DNA deletion (S1 Fig) [10]. Western blot analysis confirmed the lack of detectable HRP2 and LEDGF/p75 protein expression in MEF cells isolated from *Psip1*/*Hdgfrp2* double-deficient animals [10].

### Phenotypic characterization of knockout animals

To ascertain the affect of gene knockout on mouse development, offspring of timed heterozygous animal matings were genotyped at various time points before and/or after birth. The monitoring of 125 animals at weaning age (21 days post-birth) revealed no affect from the *Hdgfrp2* knockout on development, as the frequency of the three expected genotypes (+/+, +/g, and g/g) were indistinguishable from the predicted Mendelian frequencies of 1:2:1 (Table 1, *P* = 0.96). *Hdgfrp2* knockout mice appeared physically normal and were fertile. The 16-exon *Hdgfrp2* gene yields three mRNA isoforms that differ in the extent of exon 3 and 4 or exon 7 content [26]. The expression of all three isoforms is predictably disrupted by the utilized gene trap vector, which was inserted within the large intron between exons 3 and 4 [10, 26]. We note that mice knocked out for the namesake member of the HRP gene family, *Hdgf*, were likewise phenotypically normal [27].

**Table 1:** Genotypes of offspring from timed matings of *Hdgfrp2* heterozygous (+/g) animals.

	Number (%) of embryos				
Time point	+/+	+/g	g/g	Total	*P* value
Weaning age	30 (24.0)	64 (51.2)	31 (24.8)	125	0.96
Expected values	31 (24.8)	63 (50.4)	31 (24.8)	125	

*Psip1* knockout through gene trap vector insertion was previously reported to severely impact mouse development, as most of the mice died perinatally [16]. Embryos resulting from *Psip1* (+/-) crosses were accordingly genotyped at numerous time points including E13.5, E15.5, and E17.5. Because knockout-/- animals were recovered at or above the predictive Mendelian frequency of 25% at all three times, we conclude that the null *Psip1* allele generated via DNA deletion was not grossly deleterious to mouse embryogenesis. By contrast, only four of 302 animals reached the weaning age of 21 days (Table 2), a phenotype that is fully consistent with the perinatal lethality observed with gene trap knockout animals [16]. Histological characterization of surviving knockout mice revealed minor skeletal alterations consistent with those reported by Bickmore and colleagues (data not shown) [16]. Additional detailed histopathology of our *Psip1* knockout animals was accordingly not performed. Due to deletion of proximal exon 3 from the *Psip1* gene, we note that our genetically null animals, similar to those generated by the previously described gene trap insertion, were defective for expressing both LEDGF (p52 and p75) isoforms [15, 16].

**Table 2:** Genotypes of offspring from timed matings of *Psip1* heterozygous (+/-) animals.

	Number (%) of embryos				
Time point	+/+	+/-	-/-	Total	*P* value
E13.5	2 (8.0)	10 (40.0)	13 (52.0)	25	<0.0001
E15.5	6 (35.3)	8 (47.1)	3 (17.6)	17	0.51
E17.5	3 (27.3)	4 (36.4)	4 (36.4)	11	0.61
Weaning age	113 (37.4)	185 (61.3)	4 (1.3)	302	<0.0001
Expected values	25%	50%	25%		

Due to the perinatal lethality associated with *Psip1* knockout, we expected few if any *Psip1*/*Hdgfrp2* double knockout animals to survive to 21 days of age. Indeed, out of 322 animals, only two were genotyped as double knockout (Table 3). To ascertain when double knockout animals succumbed during development, embryos were genotyped at several time points. The percentage of double knockout animals recovered at E9.5, E11.5 and E12.5 did not significantly differ from the predicted Mendelian frequency of 12.5% (Table 3). By contrast, far fewer animals than predicted were recovered at E13.5, E14.5, and E15.5. Therefore, unlike the consequence from the sole knockout of *Psip1* [16] (Table 2), *Psip1*/*Hdgfrp2* double knockout animals succumbed during embryogenesis, at approximately E13.5.

**Table 3:** Genotypes of offspring from timed matings of *Psip1*/*Hdgfrp2* +-/+g and +-/gg animals.

	Number (%) of embryos							
Time Point	++/+g	++/gg	+-/+g	+-/gg	−−/+g	−−/gg	*n*	*P* value
E9.5	4 (6.9)	9 (15.5)	16 (27.6)	16 (27.6)	4 (6.9)	9 (15.5)	58	0.22
E11.5	8 (16.7)	8 (16.7)	8 (16.7)	11 (22.9)	5 (10.4)	8 (16.7)	48	0.19
E12.5	9 (16.1)	8 (14.3)	13 (23.2)	11 (19.6)	9 (16.1)	6 (10.7)	56	0.57
E13.5	33 (18.2)	18 (9.9)	72 (39.8)	24 (13.3)	26 (14.4)	8 (4.4)	181	0.02
E14.5	45 (19.1)	41 (17.4)	69 (29.4)	55 (23.4)	13 (5.5)	12 (5.1)	235	0.01
E15.5	10 (15.6)	8 (12.5)	22 (34.4)	19 (29.7)	3 (4.7)	2 (3.1)	64	0.04
Weaning	113 (35.1)	44 (13.7)	131 (40.7)	29 (9.0)	3 (0.9)	2 (0.6)	322	<0.0001
Expected	12.5%	12.5%	25.0%	25.0%	12.5%	12.5%		

**Fig 1:**
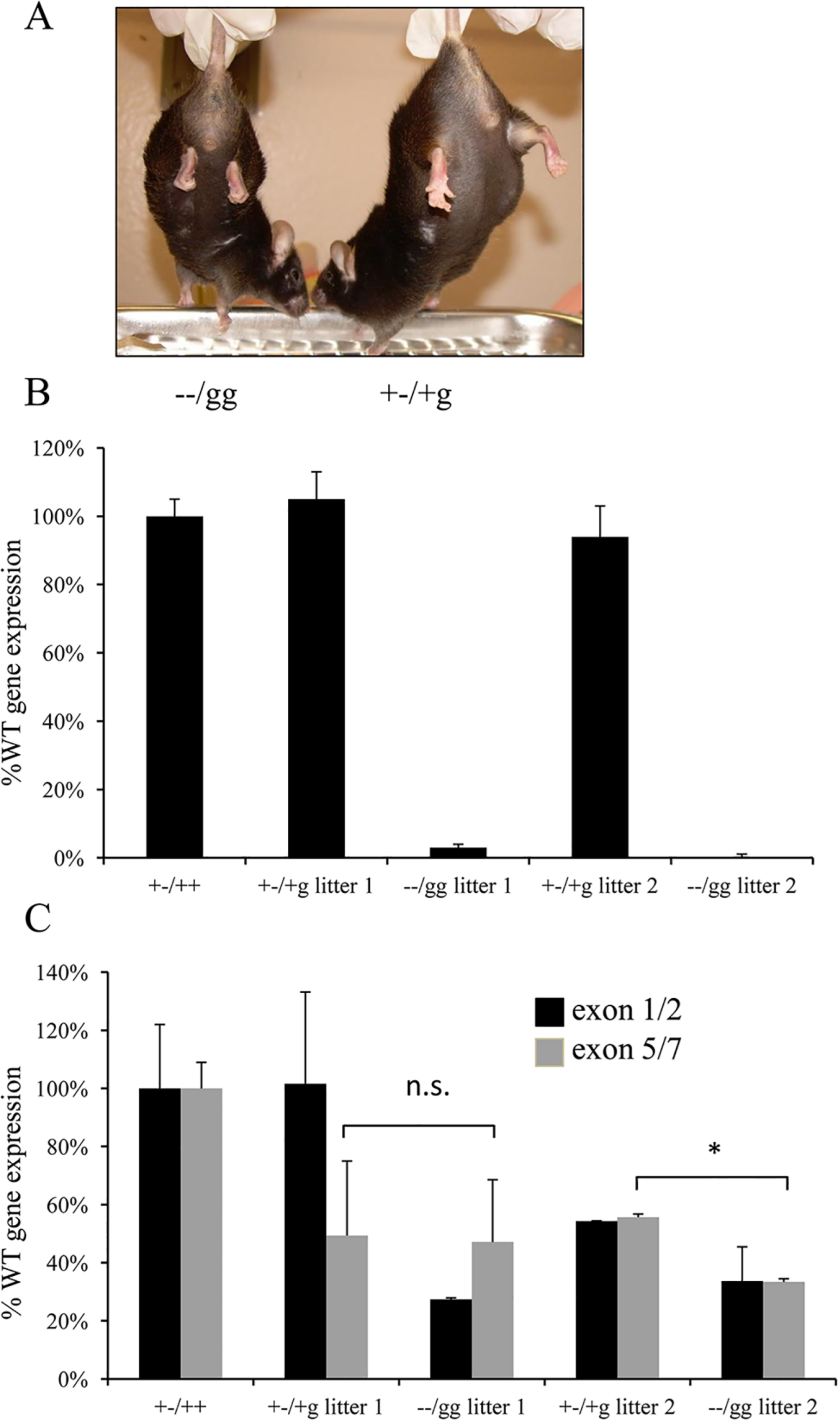
Phenotypic analysis of surviving *Psip1*/*Hdgfrp2* knockout mice. (A) The −−/gg mouse on the left, as compared to the littermate-matched heterozygous animal, revealed the tendency to clench its hind limbs. (B) Analysis of *Psip1* expression levels by qRT-PCR. (C) Levels of *Hdgfrp2* expression upstream and downstream from the gene trap insertion were detected by qRT-PCR using exon 1/2- and exon 5/7-specific primers, respectively. Heterozygous +-/+g littermate control animals for the two knockout animals (litter 1 and litter 2) and RNA from an unrelated *Psip1* heterozygous (+-/++) animal [15] served as controls in panels B and C. Panels B and C data are averages and standard deviation derived from three independent experiments. n.s., not significant; *, *P* < 0.05.

Two *Psip1*/*Hdgfrp2* double knockout mice notably survived to adulthood (Table 3). Both animals had obvious retarded movement (not shown) and the tendency to clench their hind limbs to their bodies (Fig 1A), phenotypes similar to those previously reported for *Psip1* knockout animals [16]. To test if these animals harbored *Psip1* and/or *Hdgfrp2* expression profiles that differed from those expected from dual knockout deficiency (S1 Fig), mRNA prepared from blood was compared to samples isolated from littermate matched +-/+g animals by qRT-PCR. As expected from the stable nature of DNA deletion, the *Psip1* gene expression profiles of these double knockout animals were decreased in comparison to the controls (Fig 1B). As previously noted, the expression of *Hdgfrp2* sequences downstream from the gene trap insertion in heterozygous (+-/+g) control animals, which was detected using exon 5/7-specific primers, was reduced by approximately twofold as compared to mRNA isolated from a *Hdgfrp2* +/+ control [10] (Fig 1C). The *Hdgfrp2* expression patterns of the double deficient animals differed greatly from those expected from the g/g knockout (compare Fig 1C with S1B Fig). The levels of exon 5/7-containing mRNA in the litter 1 −−/gg animal was not significantly different from its +-/+g sibling, whereas in the litter 2 animal this level was reduced < 2-fold from the matched sibling. Although this difference was statistically significant, the level of *Hdgfrp2* message distal from the gene trap in both double knockout animals matched or exceeded the level detected using upstream exon 1/2 primers (Fig 1C). We therefore suspect that mRNA read-through of the gene trap vector contributed to the survival of these two animals. The results of PCR analysis revealed that each surviving animal retained the integrated gene trap vector (not shown). Read through of the gene trap vector effectively converted the phenotype of these two animals to that of surviving *Psip1* knockout mice.

### Abnormal cardiac morphogenesis in *Psip1*/*Hdgfrp2* knockout mice

**Fig 2:**
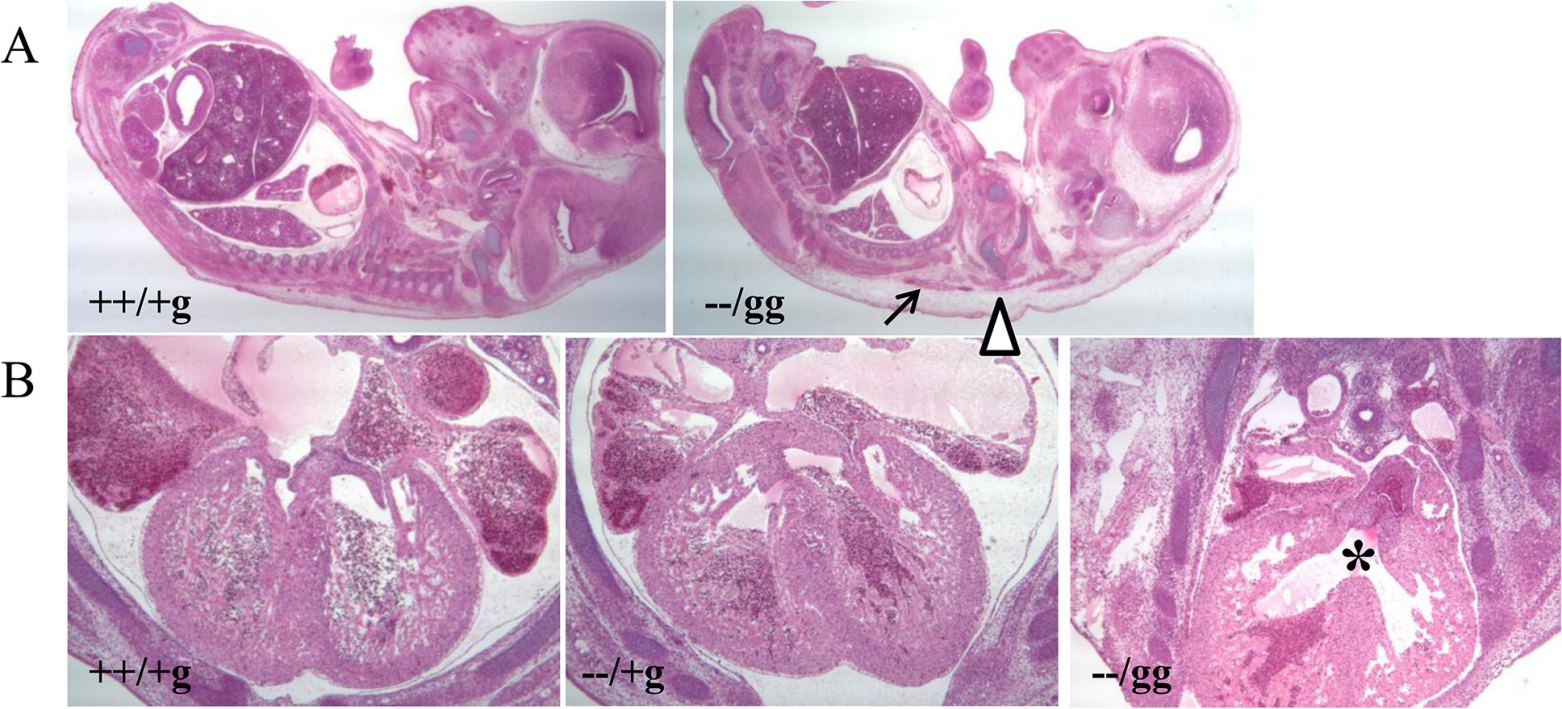
Histological analysis of control and knockout mice. (A) Sagittal section of E14.5 embryos (20X magnification). The gross morphological structures of brain, lung, liver, kidney, intestines, and other organs appeared similar between −−/gg knockout animals and littermate matched ++/+g controls (n of 4 for each genotype). Subcutaneous edema and hemorrhage in the knockout animal are marked by triangle and arrow, respectively. (B) Transverse section of littermate matched control ++/+g (left panel), *Psip1* knockout (central panel), and −−/gg double knockout (right panel) E14.5 animals (40X magnification). Normal, septated ventricles are evident in the central regions of the left and middle panels. Double knockout animals (four out of 4 analyzed) by contrast presented VSD (highlighted by an asterisk).

E14.5 embryos were histologically analyzed to investigate gross developmental defects associated with the double knockout. Serial sagittal dissection revealed subcutaneous edema and hemorrhage compared to ++/+g control animals (Fig 2A). Generalized edema is often a sign of failure in the circulatory system due to cardiac defects [28, 29]. Consistent with this, serial transverse sections demonstrated the consistent presence of VSD in double knockout but not in matched ++/+g control or *Psip1* (−−/+g) knockout animals (Fig 2B). The structures of the great aorta and pulmonary arteries by contrast appeared normal in the double knockout embryos (data not shown). The structures of other organs, such as brain, lung, liver, kidney, and intestines, also appeared normal in the double knockout animals (Fig 2A).

### Transcriptional profiling of *Psip1* and *Psip1*/*Hdgfrp2* knockout mice

To probe the molecular mechanisms that underlie the mouse knockout phenotypes, RNA extracted from heart ventricle tissue of E14.5 control ++/+g, *Psip1* (−−/+g) knockout, and double knockout embryos was subjected to RNA-Seq. The utilization of tissue from three independent sets of littermate-matched dissections afforded data filtration for biological reproducibility.

**Fig 3:**
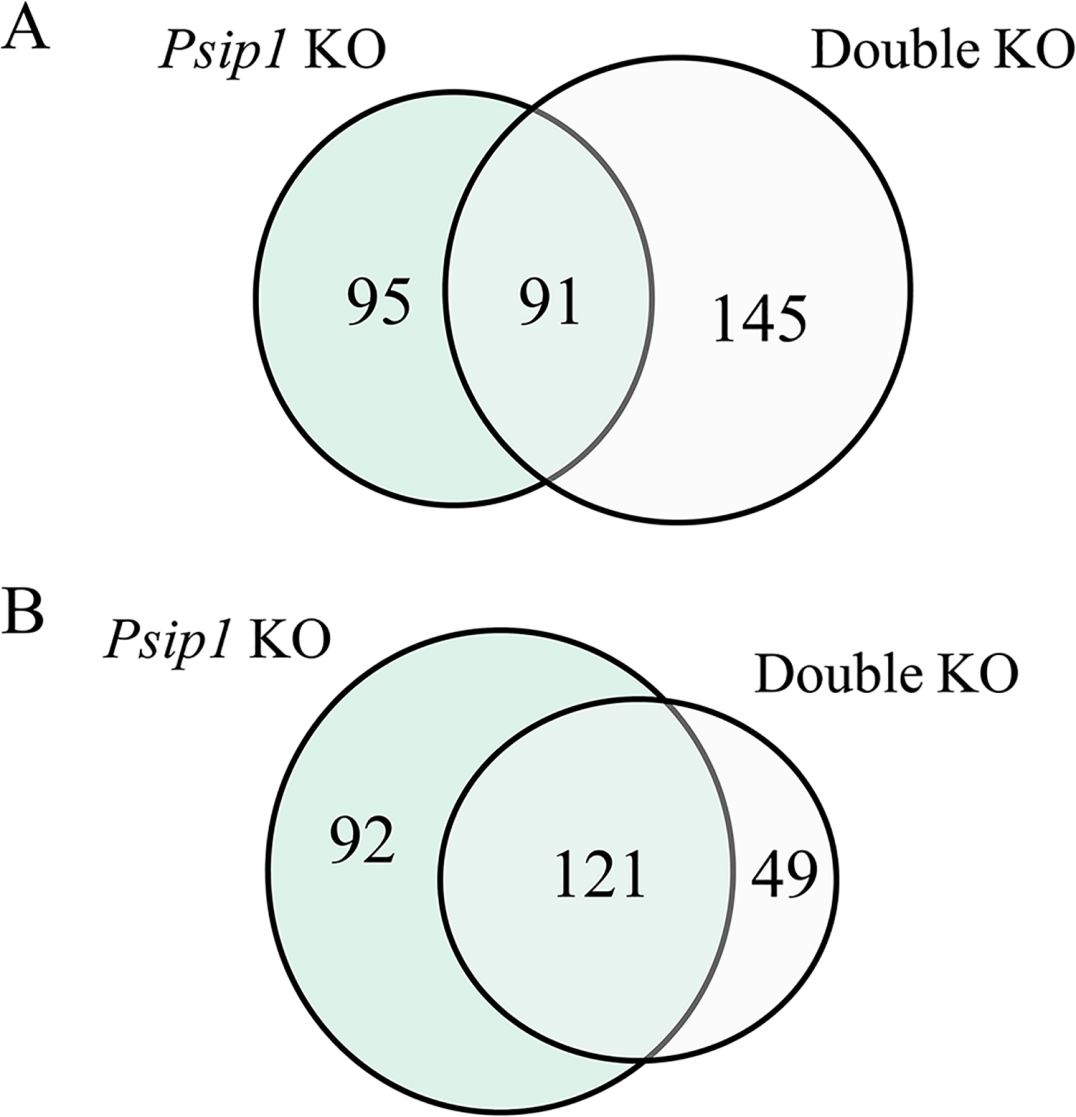
Numbers of common genes that are deregulated by *Psip1* and *Psip1*/*Hdgfrp2* knockout. (A) Venn diagram of genes up-regulated by the indicated knockout (KO) showing the overlap between conditions. The data is from Table 4. (B) Same as in (A), but for down regulated genes.

**Fig 4:**
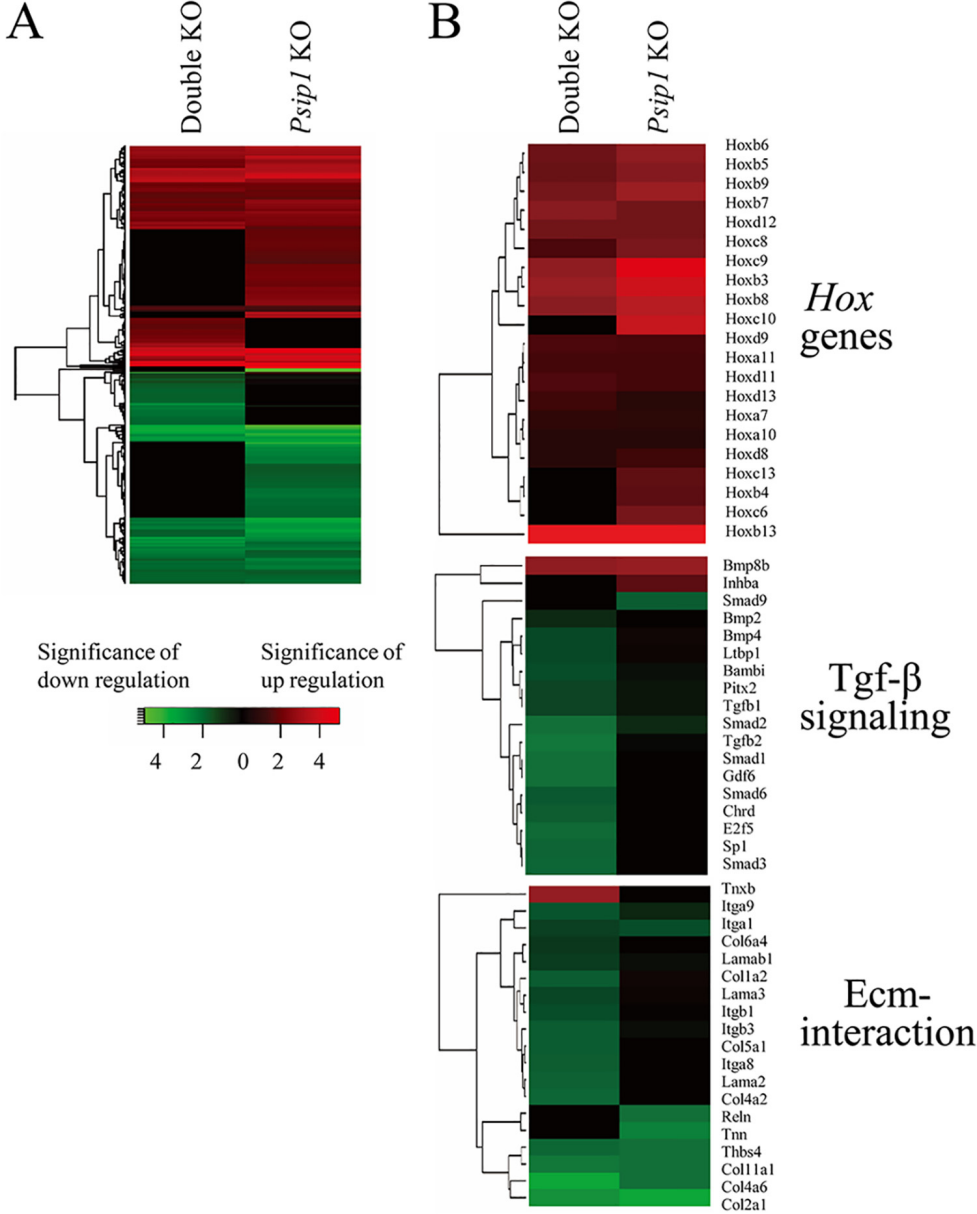
Heat maps of differentially expressed genes by pairwise comparison of knockout versus control samples. (A) Colors indicate the significance of fold change (log_2_) values in gene count level. Each row represents a gene, and each column represents the indicated comparison. Red indicates relative degree of significance of up-regulation, and green relative significance of down-regulation. Genes that failed to achieve significant levels of deregulation versus the ++/+g control were scored as zero and appear as black. (B) Differential expression patterns of *Hox* genes (upper panel) as well as genes involved in Tfg-β signaling (central panel) and the Ecm-interaction pathway (lower panel). The data were dissected out from the total gene expression profiles in panel A. KO, knockout.

A total of 12,306 protein-coding genes was detected by RNA-Seq analysis. The parsing of data in pairwise combinations revealed *Psip1* as a driving force behind differential gene expression. As shown in Table 4, 399 differentially expressed genes were determined by comparing the *Psip1* knockout and ++/+g control samples (186 up-regulated and 213 down-regulated genes), whereas 406 genes between the double knockout and control samples were differentially expressed. Because comparing the knockout samples to each other yielded 37 differentially expressed genes, the two knockout samples contained many genes in common (Fig 3). Heat map representation of the genes that were up- and down-regulated for each of the knockout conditions versus the ++/+g control highlighted several regions of overly similar differential gene expression patterns for the *Psip1* and double knockout animals (Fig 4A).

**Table 4:** Numbers of differentially expressed genes between samples.

Comparison	Total genes	Up-regulated	Down-regulated
*Psip1* KO to ++/+g	399	186	213
Double KO to ++/+g	406	236	170
*Psip1* KO to double KO	37	19	18

**K**O, knockout.

We next examined the top 20 most highly deregulated genes from the above noted pairwise comparisons (S2–S4 Tables). The expression levels of *Psip1* and *Hdgfrp2* were deregulated as expected from these comparisons. For example, *Psip1* expression was depressed about 12-fold from the control in both the *Psip1* knockout (not listed in S2 Table because it was not among the top 20 genes) and double knockout (S3 Table) samples. Similarly, *Hdgfrp2* expression was deregulated about 19-fold between the *Psip1* and double knockout samples (S4 Table). As anticipated from the results in Table 4 and Fig 3, several of the top 20 deregulated genes were identified in more than one pairwise comparison. For example, the expression levels of *Slnf2* and *Sp100* were up-regulated similarly from the comparison of *Psip1* knockout or double knockout tissue to the control sample, indicating that the *Psip1* deficiency was the likely driving factor in these expression profiles. The expression levels of several genes, including *Ddx3y*, *Kdm5d*, *Uty*, *Eif2s3y*, *Prkcq*, *Alx1*, *Enpep*, *Chrdl1*, *Rspo1*, and *Tmtc1* were moreover down-regulated in more than one pairwise comparison.

*Xist*, which plays a key role in chromosome X inactivation in mammalian somatic cells [30], was significantly upregulated by the *Psip1* knockout whereas *Ddx3y*, *Kdm5d*, *Uty*, *Eif2s3y*, each of which resides on the Y chromosome, were down-regulated significantly (S2 and S4 Tables). We accordingly wondered if the *Psip1* or double knockout influenced the sex of surviving animals. To address this five different aliquots of DNA prepared from WT, *Psip1* knockout, and double knockout heart tissue and MEF cells were monitored for the presence of the Y chromosome-specific sex-determining region Y (*Sry*) gene. Because the male-to-female ratios across all three sets of samples were 3:2 (data not shown), we conclude that, given these somewhat modest sample sizes, neither the *Psip1* nor *Psip1*/*Hdgfrp2* knockout significantly influenced animal gender. The *Psip1* knockout samples subjected to RNA-Seq were derived from one male and two female embryos, while the double knockout and WT control (++/+g) samples were each derived from two male and one female animal. We accordingly suspect that this accounts for the significant levels of deregulated X- and Y-chromosome genes observed in the *Psip1* versus double knockout or WT pairwise combinations (S2–S4 Tables).

Numerous cellular proteins, including Jpo2 [31, 32], Pogz [33], Menin [34], Dbf4/Ask [35], Mll [36], and Iws1 [37] interact with LEDGF/p75 through the integrase-binding domain while other factors, including Tox4, Nova1, Mcm7, C3orf59, and Map1a, interact with the PWWP domain that is in common to both LEDGF/p75 and LEDGF/p52 [38]. The genes that encode known LEDGF-interacting proteins were queried to ascertain if either the *Psip1* knockout or *Psip1*/*Hdgfrp2* double knockout altered their expression levels in embryonic heart tissue. *Nova1*, whose expression was up-regulated approximately threefold by both knockout conditions, was the only gene among this set that scored as significantly deregulated (S5 Table). Because Nova1 is an RNA splicing factor, the expression levels of 138 additional genes that were identified from using the gene ontology search term “mRNA splicing, via spliceosome”, which included the *Sfrs1* gene that encodes for the LEDGF/p52-interacting protein ASF/SF2 (see below), were queried. The only other gene with deregulated expression among the expanded set of RNA splicing factors was *Psip1*.

RT-PCR was utilized to confirm the expression profiles of a subset of genes that were determined as differentially regulated by RNA-Seq. For example, significant up-regulation of *Slfn* expression was confirmed in both the *Psip1* and double knockout samples (about 11-fold in each), though these values were tampered somewhat from the approximate 48- and 18-fold levels of up-regulation determined by RNA-Seq for the *Psip1* knockout and double knockout samples, respectively (S2 and S3 Tables). Extending this analysis to a set of seven genes that were deregulated to milder levels (from ~20% to 5-fold; S2A Fig) confirmed the deregulated gene expression profiles that were detected by RNA-Seq (S2 Fig, compare panels A and B).

Bickmore and colleagues previously noted that *Psip1* knockout significantly deregulated the expression of numerous homeobox (*Hox*) genes [16, 39], a result that was generally confirmed here (S2 and S6 Tables; Fig 4B). The expression of the *Hoxb13* gene was most significantly up-regulated, by ~300 to 400-fold, by both *Psip1* knockout and double knockout when compared to matched ++/+g controls. The expression levels of *Hoxa1* and *Hoxa3*, which from the RNA-Seq analysis were not significantly deregulated by the knockouts, as well as *Hoxb3* and *Hoxc9*, which were up-regulated by ~7 to 17-fold (S6 Table), were queried by qRT-PCR. For this analysis, RNA derived from embryonic head and limb tissue was additionally compared to heart-derived RNA. While the expression levels of *Hoxa1* and *Hoxa3* were not significantly altered by the knockouts, the expression levels of *Hoxb3* and *Hoxc9* were significantly up-regulated, by ~4 to 10-fold, in all tissues examined from knockout animals (S3A Fig). We also confirmed significant levels of up-regulation of *Hoxb3* and *Hoxb13* expression in MEF cells derived from *Psip1* and double knockout animals (S3B Fig).

### Gene ontology and pathway analyses of differentially expressed genes

Ontology term analysis of the genes that were differentially expressed between the double knockout and control samples revealed statistically significant differences in anatomical structure development, cell differentiation, proteinaceous Ecm, extracelluar region, and cell adhesion (S4A Fig). For the comparison between *Psip1* knockout and control, proteinaceous Ecm, extracelluar region, cell adhesion, extracellular space, and nucleic acid binding transcription factor activity were significantly enriched among the differentially expressed genes (S4B Fig).

The GAGE R package was utilized to analyze gene set and KEGG pathways taking into account all differentially expressed genes and the associated fold change values [20]. Significantly regulated KEGG pathways (q-value < 0.1) are reported in Table 5. The comparison of *Psip1* knockout and control ++/+g samples did not yield a significantly regulated pathway. By contrast, several pathways emerged from comparing the double knockout and control samples: ribosome, ribosome biogenesis in eukaryotes, and RNA transport were up-regulated, whereas Tgf-β signaling, protein digestion and absorption, focal adhesion, Ecm-receptor interaction, and lysosome were down regulated. Comparing the double knockout and *Psip1* knockout samples yielded the sole down regulated pathway of Tgf-β signaling (Table 5).

**Table 5:** Significantly deregulated metabolic pathways across samples.

Comparison	Up-regulated	q-value	Down-regulated	q-value
*Psip1* KO vs. ++/+g	n.a.	n.a.	n.a.	n.a.
Double KO vs. ++/+g	Ribosome biogenesis in eukaryotes	0.006	Tgf-β pathway	0.04
	RNA transport	0.006	Protein digestion and absorption	0.03
	Ribosome	0.013	Ecm-receptor interaction	0.03
			Focal adhesion	0.01
			Lysosome	0.01
Double vs. *Psip1* KO	n.a.	n.a.	Tgf-β pathway	0.03

**K**O, knockout; n.a., not applicable.

**Fig 5:**
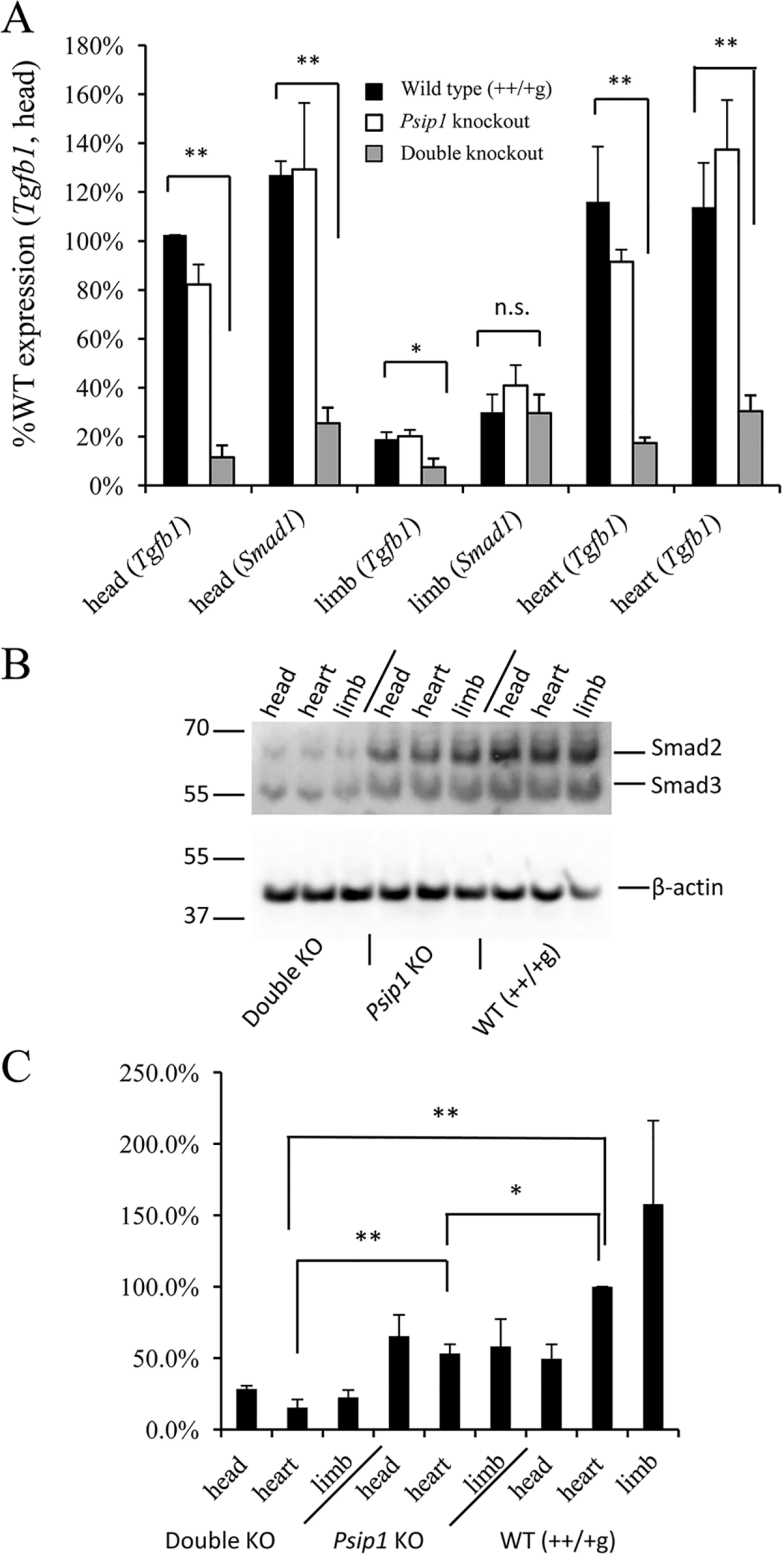
*Tgfb1* and *Smad* expression profiles. (A) RT-PCR analysis of *Tgfb1* and *Smad1* expression (average and standard deviation from two independent sets of qRT-PCR measurements). *Tgfb1* and *Smad1* expression levels in the double knockout samples were statistically different from the matched ++/+g controls in all tissues tested with the exception of *Smad1* expression in embryonic limb tissue. n.s., not significant. (B) Western blot of Smad 2/3 protein levels in the indicated tissues. The migration positions of mass standards in kDa are indicated to the left; β-actin was blotted as a protein loading control. (C) Quantification of Smad2/3 proteins normalized for β-actin content for n = 3 independent experiments (average and standard deviation). *, *P* < 0.05; **, *P* < 0.01.

The Tgf-β signaling pathway regulates different processes related to cardiovascular biology, including cardiac development and angiogenesis [40, 41]. Knockout of the *Tgfb1* gene is embryonic lethal to mice due to inflammation of the heart and lungs [40, 42] and *Tgfb2* knockout is embryonic lethal due in part to VSD, myocardial thinning, and a double outlet right ventricle [43]. Pathview visualization revealed significant deregulation of several key genes in the Tgf-β signaling pathway including *Tgfb1*, *Bmp* [44], *Activin* [45], and *Smad* [46] (S5 Fig). To confirm these results, *Tgfb1* and *Smad1* expression levels were analyzed by qRT-PCR utilizing RNA extracted from embryonic head, limb, and heart tissues. The expression level of *Tgfb1* was significantly down regulated across the tissues derived from double knockout animals, while *Smad1* expression was significantly down regulated in head and in heart tissue (Fig 5A). Western blot analysis confirmed lower levels of Smad2/3 protein expression in the tissues derived from the double knockout as compared to *Psip1* knockout and control animals (Fig 5B and 5C).

Heart development depends on the interaction between integrins in cardiomyocytes with the surrounding Ecm [47] and Tgf-β signaling influences the expression of several integrins [48–50] and the production of the Ecm [51]. The knockout of β1 integrin moreover resulted in embryonic lethality with associated cardiac defects [52–54]. We accordingly speculate that down-regulation of the Ecm receptor interaction pathway could result from the down-regulation of the TGF-β signaling pathway. Focal adhesion kinase (FAK) is a key downstream mediator of Ecm-integrin signaling [55] and FAK knockout mice die within hours of birth due to incomplete formation of the septum between the ventricles and overriding aorta [56]. Down regulation of the focal adhesion pathway in our embryonic tissue samples is consistent with a role for TGF-β signaling in the underlying cardiac pathology and prenatal lethality of the double knockout *Psip1*/*Hdgfrp2* mice.

The transcriptional activities of Hox proteins can be regulated by Smads [57]. Group 13 Hox proteins Hoxa13 and Hoxd13 in particular were reported to interact with Smad5 and repressed Smad-mediated transcriptional activation [58]. Although this prior study did not study Hoxb13, it is tempting to speculate that the significant up-regulation of Hoxb13 observed here could have negatively impacted *Smad* gene expression and Tgf-β signaling. If true, other factors, which would be attributable to the *Hdgfrp2* knockout, must contribute to the VSD, as the Tfg-β signaling pathway was not deregulated through sole *Psip1* knockout (Table 5) yet *Hoxb13* expression was similarly up-regulated under both knockout conditions (S6 Table and S3B Fig).

LEDGF/p52 can interact with the mRNA splicing factor ASF/SF2 (the product of the *Sfrs1* gene) and modulate its activity [59, 60], and ASF/SF2 has been identified as a key participant in regulated postnatal heart remodeling in mice [61]. To ascertain if *Psip1* knockout resulted in differential mRNA splicing in heart tissue, the RNA-Seq data was analyzed using multivariate analysis of transcript splicing (MATS) [62]. No significant alternative splicing events were detected by comparing the *Psip1* knockout and ++/+g control datasets. Comparing the double knockout and control samples yielded five alternative splicing events (S7 Table). The expression level of the *Sfrs1* gene, which encodes for ASF/SF2, was not significantly deregulated in *Psip1* knockout (*P* = 0.64) or *Psip1*/*Hdgfrp2* double knockout (*P* = 0.69) embryonic ventricular tissue compared to the matched ++/+g control samples (S5 Table). Although we cannot rule out a role for mRNA splicing in the VSD, the lack of significant deregulation of RNA splicing factor gene expression, which was detected for only *Nova1* and *Psip1* among a set of 139 genes, leads us to believe that differential splicing was not a driving factor in the underlying pathology of VSD.

## Conclusions

Our genetically null *Psip1* knockout mice phenocopied the perinatal lethality previously reported using gene trap vector insertion [16]. While knockout of the related HRP2 protein product did not detectably influence mouse development, *Psip1*/*Hdgfrp2* double deficiency resulted in embryonic lethality at approximate E13.5 with associated VSD. RNA-Seq analysis revealed significant deregulation of the Tgf-β signaling pathway as well as deregulation of downstream Ecm-interaction and focal adhesion pathways in the tissue of double knockout animals. The expression levels of genes that encode for key LEDGF/p75 interacting proteins, such as Menin and Mll, were not altered by the knockouts described here. Though the expression level of the *Nova1* gene, which encodes for an RNA splicing factor that interacts with LEDGF/p75, was up-regulated, the expression levels of genes that encode for other RNA splicing factors, including the key LEDGF/p52 interactor ASF/SF2, were not altered significantly. We conclude that the deregulation the Tgf-β signaling pathway was a likely contributing factor to abnormal cardiac morphogenesis and prenatal mortality of the *Psip1*/*Hdgfrp2* double-deficient mice.

## Supporting Information

S1 Fig Genotypic and phenotypic characterization of animals generated during the double knockout mating scheme. (A) Genomic DNA from tails of mouse embryos were subjected to PCR using the indicated primer pairs. Primers AE2331 and AE2802 detect a 803 bp product from wild-type *Psip1* DNA whereas exon 3 deletion yields a 324 bp fragment [15]. The 535 bp *Hdgfrp2* product (primers AE2511, AE2512) is disrupted by gene trap insertion; primers AE3747 and AE3748, specific for the pGT2lfx vector, detect a 433 bp product in all cell types [10]. The migration positions of expected DNA products in bp and of mass standards in kb are shown to the left and right sides of the gels, respectively. DNA was detected by ethidium bromide staining. (B) Phenotypic characterization of *Hdgfrp2* mRNA expression levels using the indicated primer pairs. *P* values from the indicated comparisons of expression levels are shown. (C) *Psip1* mRNA expression levels. The data in panels B and C are averages and standard deviation of three independent experiments, with qRT-PCR samples conducted in duplicate for each experiment.
(PDF)

S2 Fig RT-PCR analysis of a subset of genes that were detected by RNA-Seq as differentially regulated by *Psip1* and/or *Psip1*/*Hdgfrp2* knockout. (A) RNA-Seq data expressed as log_2_ fold changes in mRNA expression levels with associated *P* values for the seven indicated genes. (B) Results from qRT-PCR analysis (average and standard deviation from three independent sets of qRT-PCR measurements). The levels of gene expression in the double knockout samples in panel B were statistically different (*P* < 0.05) from the matched ++/+g controls for all seven genes whereas the levels of expression of only two genes in the *Psip1* knockout samples, Integrin α1 and Caveolin 2, achieved significance versus the controls. n.s., not significant (control versus *Psip1* knockout comparison).
(PDF)

S3 Fig RT-PCR analysis of *Hox* gene expression. (A) Results from qRT-PCR analysis of the indicated *Hox* genes in different embryonic tissue (average and standard deviation from two independent sets of qRT-PCR measurements). *Hoxb3* and *Hoxc9* gene expression levels in the double knockout and *Psip1* knockout samples were statistically different from the matched ++/+g controls across tissues, while *Hoxa1* and *Hoxa3* gene expression levels were not. (B) Levels of *Hoxb3* and *Hoxb13* expression in *Psip1* and *Psip1*/*Hdgfrp2* knockout MEFs. The expression of both *Hoxb3* and *Hoxb13* was significantly up-regulated (average and standard deviation from two independent sets of qRT-PCR measures). **, *P* < 0.01.
(PDF)

S4 Fig Enriched gene ontology terms between expression patterns in control and knockout ventricular tissue. (A) Comparison of double knockout (−−/gg) versus control samples. (B) *Psip1* knockout versus control.
(PDF)

S5 Fig KEGG analysis of differential gene expression in the Tgf-β signaling pathway. The data is from the comparison of double knockout and ++/+g control samples. Symbol codes: +p, phosphorylation;-p, dephosphorylation; +u, ubiquitination; → o →, activation; –**|** o →, repression. Dashed lines indicate indirect effects. The heat map key indicates relative degrees of significance in levels of transcriptional down-regulation (green) and up-regulation (red).
(PDF)

S1 Table Sequences of PCR primers. (PDF)

S2 Table Top 20 differentially expressed genes comparing *Psip1* knockout to control ++/+g tissue. (PDF)

S3 Table Top 20 differentially expressed genes comparing *Psip1*/*Hdgfrp2* knockout to control ++/+g tissue. (PDF)

S4 Table Top 20 differentially expressed genes comparing *Psip1* knockout to *Psip1*/*Hdgfrp2* knockout tissue. (PDF)

S5 Table Gene expression profiles of LEDGF interacting proteins vs. ++/+g control. (PDF)

S6 Table Differential expression of *Hox* genes in pairwise tissue comparisons. (PDF)

S7 Table Alternatively spliced genes in double knockout versus control ++/+g samples. (PDF)
