# Supplementary material for: Embryonic Lethality Due to Arrested Cardiac Development in Psip1/Hdgfrp2 Double-Deficient Mice
Source: PLoS One. 2015 Sep 14;10(9):e0137797. doi: 10.1371/journal.pone.0137797 (PMC4569352; doi:10.1371/journal.pone.0137797)
Supplement: S2 Fig — (A) RNA-Seq data expressed as log2 fold changes in mRNA expression levels with associated P values for the seven indicated genes. (B) Results from qRT-PCR analysis (average and standard deviation from three independent sets of qRT-PCR measurements). The levels of gene expression in the double knockout samples in panel B were statistically different (P < 0.05) from the matched ++/+g controls for all seven genes whereas the levels of expression of only two genes in the Psip1 knockout samples, Integrin α1 and Caveolin 2, achieved significance versus the controls. n.s., not significant (control versus Psip1 knockout comparison). (PDF) [file pone.0137797.s002.pdf]

A

	Double KO vs. ++/+g		*Psip1* KO vs. ++/+g	
**Gene**	**log_2 fold change**	***P* value**	**log_2 fold change**	***P* value**
Tgf-β1	-1.33	0.009	-0.34	0.5
Smad1	-2.2	<0.001	-0.81	0.11
Integrin α1	-1.28	0.014	-1.51	0.004
Integrin α9	-1.64	0.002	-0.72	0.16
Pik3r5	-1.53	0.02	-0.34	0.74
E2f5	-2.06	<0.001	0.91	0.08
Caveolin 2	-1.9	<0.001	-1.3	0.01

KO, knockout

B

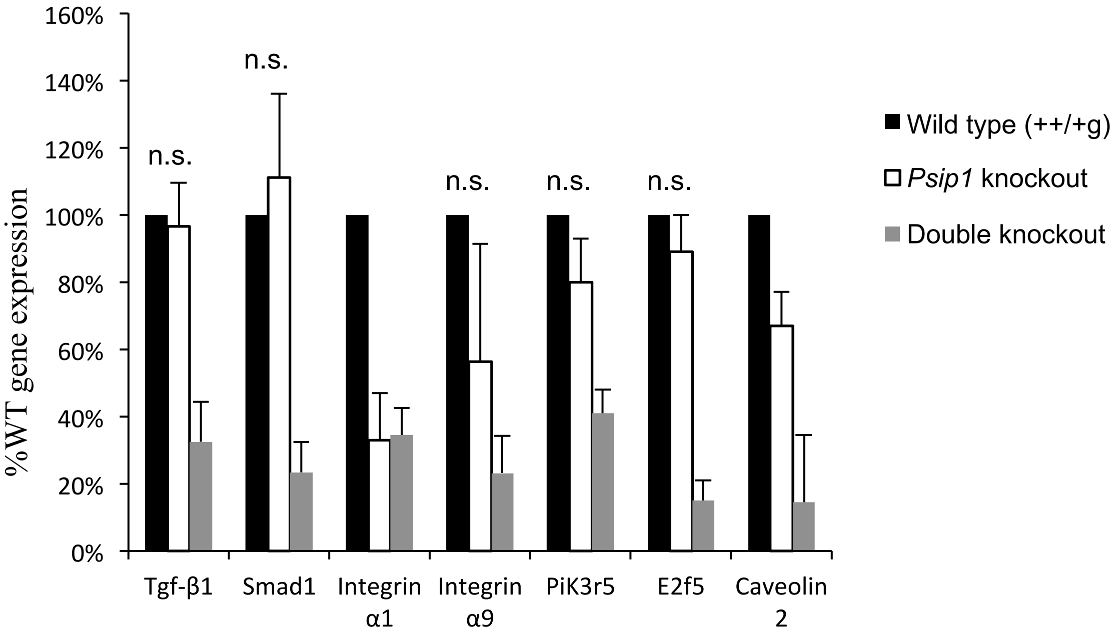

S2 Figure
