# Supplementary figures and images for: Embryonic Lethality Due to Arrested Cardiac Development in Psip1/Hdgfrp2 Double-Deficient Mice
Source: PLoS One. 2015 Sep 14;10(9):e0137797. doi: 10.1371/journal.pone.0137797 (PMC4569352; doi:10.1371/journal.pone.0137797)

A

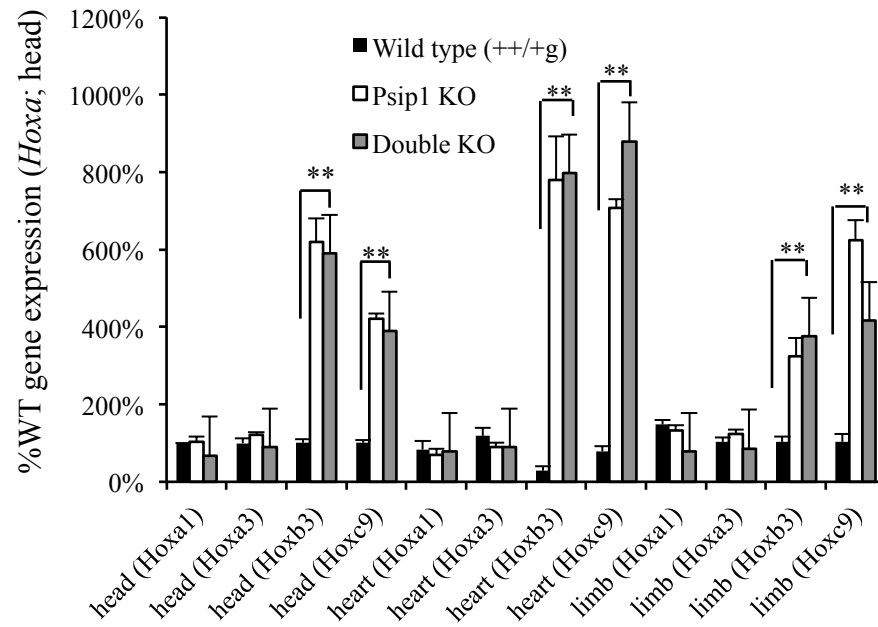

B

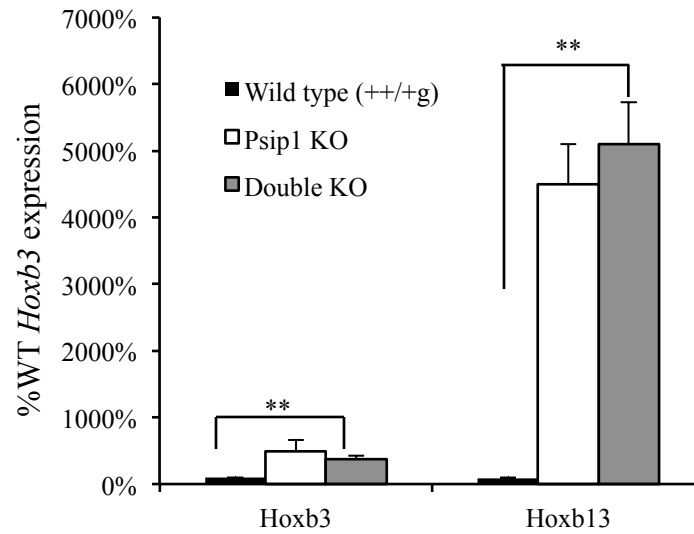

S3 Figure

Supplement: S3 Fig — (A) Results from qRT-PCR analysis of the indicated Hox genes in different embryonic tissue (average and standard deviation from two independent sets of qRT-PCR measurements). Hoxb3 and Hoxc9 gene expression levels in the double knockout and Psip1 knockout samples were statistically different from the matched ++/+g controls across tissues, while Hoxa1 and Hoxa3 gene expression levels were not. (B) Levels of Hoxb3 and Hoxb13 expression in Psip1 and Psip1/Hdgfrp2 knockout MEFs. The expression of both Hoxb3 and Hoxb13 was significantly up-regulated (average and standard deviation from two independent sets of qRT-PCR measures). **, P < 0.01. (PDF) [file pone.0137797.s003.pdf]

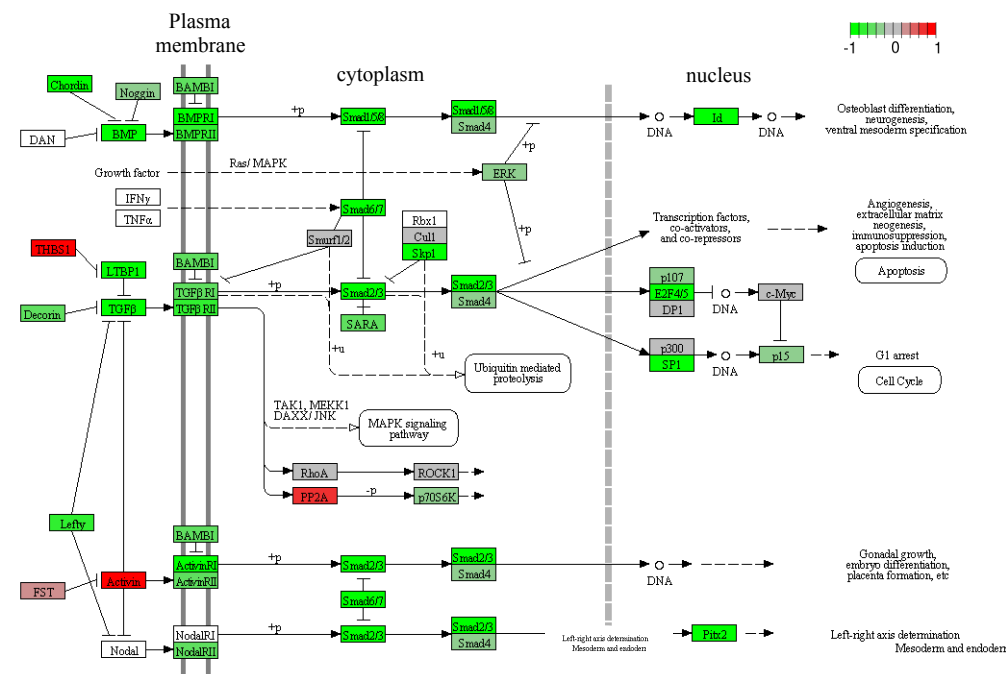

S5 Figure

Supplement: S5 Fig — The data is from the comparison of double knockout and ++/+g control samples. Symbol codes: +p, phosphorylation;-p, dephosphorylation; +u, ubiquitination; → o →, activation; –| o →, repression. Dashed lines indicate indirect effects. The heat map key indicates relative degrees of significance in levels of transcriptional down-regulation (green) and up-regulation (red). (PDF) [file pone.0137797.s005.pdf]
