# Supplementary material for: Embryonic Lethality Due to Arrested Cardiac Development in Psip1/Hdgfrp2 Double-Deficient Mice
Source: PLoS One. 2015 Sep 14;10(9):e0137797. doi: 10.1371/journal.pone.0137797 (PMC4569352; doi:10.1371/journal.pone.0137797)
Supplement: S4 Fig — (A) Comparison of double knockout (−−/gg) versus control samples. (B) Psip1 knockout versus control. (PDF) [file pone.0137797.s004.pdf]

A

**Double knockout vs. ++/+g control**	***P* value**
Anatomical structure development	5×10^{-17}
Cell differentiation	6×10^{-13}
Proteinaceous extracellular matrix	2×10^{-12}
Extracellular region	6×10^{-10}
Cell adhesion	2×10^{-9}

B

***Psip1* knockout vs. ++/+g control**	***P* value**
Proteinaceous extracellular matrix	6×10^{-18}
Extracellular region	3×10^{-15}
Cell adhesion	2×10^{-12}
Extracellular space	9×10^{-10}
Nucleic acid binding transcription factor activity	7×10^{-10}

S4 Figure
