# Supplementary material for: Embryonic Lethality Due to Arrested Cardiac Development in Psip1/Hdgfrp2 Double-Deficient Mice
Source: PLoS One. 2015 Sep 14;10(9):e0137797. doi: 10.1371/journal.pone.0137797 (PMC4569352; doi:10.1371/journal.pone.0137797)
Supplement: S1 Table — (PDF) [file pone.0137797.s006.pdf]

S1 Table. Sequences of PCR primers.

Primer	Sequence (5'-3')	Purpose
AE2331	GAGATATCGAGGCAGAAAGAAGACTGGGATAG	*Psip1* genotyping
AE2802	GCATGGTGGCACAATGGCAACTGGGTC	*Psip1* genotyping
AE2511	TTCTGTTCTCATCCATGCTCTAGTG	*Hdgfrp2* genotyping
AE2512	GCTGTTTATTGGCTTGCTAACCATG	*Hdgfrp2* genotyping
AE3747	GTTGCGCAGCCTGAATGGCG	*Hdgfrp2* genotyping
AE3748	GCCGTCACTCCAACGCAGCA	*Hdgfrp2* genotyping
AE6796	TTGTCTAGAGAGCATGGAGGGCCATGTCAA	*Sry* genotyping
AE6797	CCACTCCTCTGTGACACTTTAGCCCTCCGA	*Sry* genotyping
AE2624	CCTCAAACATGACTCGCGATTTC	*Psip1* expression
AE2625	GCTCCATCAGGAACTTCATCTAC	*Psip1* expression
AE3160	TCCGACTCCCGCCTCTGACTT	*Hdgfrp2* expression
AE3161	CACGGCACCATCAGCAATGTC	*Hdgfrp2* expression
AE2553	TGAGTCGGAGAAGACCAGTGACC	*Hdgfrp2* expression
AE2554	AATCCGAGGCTGATGGCACCTTC	*Hdgfrp2* expression
AE3664	AGGTCCTGGCATCTTGTCCATGG	*Ppia* expression
AE3665	GGCTTCCACAATGTTCATGCC	*Ppia* expression
AE6770	CCTTCCCTCGGATGTGAGTC	*Itga1* expression
AE6771	AAGTTCTCCCCGTATGGTAAGA	*Itga1* expression
AE6772	CTCCCGTGGCTTCTAGTGC	*Tgfb1* expression
AE6773	GCCTTAGTTTGGACAGGATCTG	*Tgfb1* expression
AE6774	GCTTCGTGAAGGGTTGGGG	*Smad1* expression
AE6775	CGGATGAAATAGGATTGTGGGG	*Smad1* expression
AE6776	AAGTGTCGTGTCCATACCAAC	*Itga9* expression
AE6777	GGTCTGCTTCGTAGTAGATGTTC	*Itga9* expression
AE6778	TGCTCTGGAGCGATGCTTG	*Pik3r5* expression
AE6779	ACCTCTTGGGTCTTTTGTAGGA	*Pik3r5* expression
AE6780	CCACCAAATTCGTGTCGTTGC	*E2f5* expression
AE6781	AGCACCTACACCCTTCCACT	*E2f5* expression
AE6782	TCACCAGCTCAACTCTCATCT	*Cav2* expression
AE6783	GCCAGAAATACGGTCAGGAACT	*Cav2* expression
AE6784	ATGGGTACTAGACTTGAGGCAA	*Slfn2* expression
AE6785	AGCTTAGCATATTTGGCTTCCAG	*Slfn2* expression
AE6786	ACAGAACTTCAGTGCGCCTTACA	*Hoxa1* expression
AE6787	AGACCC GGGAGCGACAGGCTTCTTG	*Hoxa1* expression
AE6788	CCTGGATGAAAGAGTCTCGACAA	*Hoxa3* expression
AE6789	CAG GCCAGCGCAGCTTTCG	*Hoxa3* expression
AE6790	TCACCCAGCGATGCAGAA	*Hoxb3* expression
AE6791	CGAGGAATAGCCTCCGAAGA	*Hoxb3* expression
AE6792	GCTGATGCCTGCTGTCAACTAT	*Hoxb13* expression
AE6793	ATTGCTTTGGCGGCTCC	*Hoxb13* expression
AE6794	TCATCCTTCGATTCTGAAACCA	*Hoxc9* expression
AE6795	GGTGGCCCGGGTTCTC	*Hoxc9* expression
