# Supplementary material for: Embryonic Lethality Due to Arrested Cardiac Development in Psip1/Hdgfrp2 Double-Deficient Mice
Source: PLoS One. 2015 Sep 14;10(9):e0137797. doi: 10.1371/journal.pone.0137797 (PMC4569352; doi:10.1371/journal.pone.0137797)
Supplement: S2 Table — (PDF) [file pone.0137797.s007.pdf]

S2 Table. Top 20 differentially expressed genes comparing *Psip1* knockout to control ++/+g tissue.

Symbol	Gene name	log2 fold change
Xist	Inactive X specific transcripts	11.13
Sprr2k	Small proline-rich protein 2K	8.50
Hoxb13	Homeobox B13	8.29
AI662270	Expressed sequence AI662270	8.08
Slfn2	Schlafen 2	5.60
Trank1	Tetratricopeptide repeat and ankyrin repeat containing 1	4.87
Hoxc9	Homeobox C9	4.37
Sp100	Nuclear antigen Sp100	4.09
Ddx3y	DEAD (Asp-Glu-Ala-Asp) box polypeptide 3	-11.00
Kdm5d	Lysine (K)-specific demethylase 5D	-9.21
Uty	Ubiquitously transcribed tetratricopeptide repeat gene	-8.90
Eif2s3y	Eukaryotic translation initiation factor 2	-8.64
Prkcq	Protein kinase C	-6.01
Alx1	ALX homeobox 1	-4.66
Enpep	Glutamyl aminopeptidase	-4.48
Chrdl1	Chordin-like 1	-4.28
Cldn1	Claudin 1	-4.19
Rspo1	R-spondin homolog (Xenopus laevis)	-4.09
Tmtc1	Transmembrane and tetratricopeptide repeat containing 1	-3.98
Gfra2	Glial cell line derived neurotrophic factor family receptor alpha 2	-3.83
