# Supplementary material for: Embryonic Lethality Due to Arrested Cardiac Development in Psip1/Hdgfrp2 Double-Deficient Mice
Source: PLoS One. 2015 Sep 14;10(9):e0137797. doi: 10.1371/journal.pone.0137797 (PMC4569352; doi:10.1371/journal.pone.0137797)
Supplement: S3 Table — (PDF) [file pone.0137797.s008.pdf]

S3 Table. Top 20 differentially expressed genes comparing *Psip1/Hdgfrp2* knockout to control ++/+g tissue.

Symbol	Gene name	log2 fold change
Hoxb13	Homeobox B13	8.55
Gda	Guanine deaminase	4.98
Sult4a1	Sulfotransferase family 4A,member 1	4.58
Zic1	Zinc finger protein of the cerebellum 1	4.24
Slfn2	Schlafen 2	4.17
Cntn1	Contactin 1	3.78
Krt19	Keratin 19	3.61
Sp100	Nuclear antigen Sp100	3.50
Slc26a7	Solute carrier family 26, member 7	-4.91
Sorbs2	Sorbin and SH3 domain containing 2	-3.96
Chrdl1	Chordin-like 1	-3.92
Tmtc1	Transmembrane and tetratricopeptide repeat containing 1	-3.71
Perp	PERP, TP53 apoptosis effector	-3.69
Alx1	ALX homeobox 1	-3.59
Prkcq	Protein kinase C, theta	-3.57
Enpep	Glutamyl aminopeptidase	-3.55
Pax9	Paired box 9	-3.49
Rspo1	R-spondin homolog (Xenopus laevis)	-3.47
Psip1	PC4 and SFRS1 interacting protein 1	-3.40
Clec11a	C-type lectin domain family 11, member a	-3.23
