# Supplementary material for: Embryonic Lethality Due to Arrested Cardiac Development in Psip1/Hdgfrp2 Double-Deficient Mice
Source: PLoS One. 2015 Sep 14;10(9):e0137797. doi: 10.1371/journal.pone.0137797 (PMC4569352; doi:10.1371/journal.pone.0137797)
Supplement: S4 Table — (PDF) [file pone.0137797.s009.pdf]

S4 Table. Top 20 differentially expressed genes comparing *Psip1* knockout to *Psip1/Hdgfrp2* knockout tissue.

Symbol	Gene name	log2 fold change
Xist	Inactive X specific transcripts	12.56
Slc26a7	Solute carrier family 26, member 7	4.31
Hdgfrp2	Hepatoma-derived growth factor related protein 2	4.23
AI662270	Expressed sequence AI662270	3.70
Trp63	Transformation related protein 63	3.04
E2f5	E2F transcription factor 5	2.97
C1qtnf7	C1q and tumor necrosis factor related protein 7	2.40
Chodl	Chondrolectin	2.37
Ddx3y	DEAD (Asp-Glu-Ala-Asp) box polypeptide 3, Y-linked	-10.77
Kdm5d	Lysine (K)-specific demethylase 5D	-9.33
Uty	Ubiquitously transcribed tetratricopeptide repeat gene, Y chromosome	-8.75
Eif2s3y	Eukaryotic translation initiation factor 2, subunit 3, structural gene Y-linked	-8.74
Clec3b	C-type lectin domain family 3, member b	-4.27
Serpina3n	Serine (or cysteine) peptidase inhibitor, clade A, member 3N	-3.52
Mdk	Midkine	-3.36
Comp	Cartilage oligomeric matrix protein	-2.96
Clec14a	C-type lectin domain family 14, member a	-2.72
Gda	Guanine deaminase	-2.64
Scg2	Secretogranin II	-2.52
Fgl2	Fibrinogen-like protein 2	-2.24
