# Supplementary material for: Embryonic Lethality Due to Arrested Cardiac Development in Psip1/Hdgfrp2 Double-Deficient Mice
Source: PLoS One. 2015 Sep 14;10(9):e0137797. doi: 10.1371/journal.pone.0137797 (PMC4569352; doi:10.1371/journal.pone.0137797)
Supplement: S5 Table — (PDF) [file pone.0137797.s010.pdf]

S5 Table. Gene expression profiles of LEDGF interacting proteins vs. ++/+g control.

Gene	Interactor	Comparator	Log_2 fold change	*P* value
Jpo2	LEDGF/p75	Psip1 KO	-0.07	0.89
		Double KO	0.28	0.58
PogZ	LEDGF/p75	Psip1 KO	0.02	0.98
		Double KO	-0.02	0.97
Men1	LEDGF/p75	Psip1 KO	0.06	0.91
		Double KO	0.07	0.89
CtIP	LEDGF/p75	Psip1 KO	-0.07	0.89
		Double KO	-0.06	0.91
Mll1	LEDGF/p75	Psip1 KO	0.14	0.78
		Double KO	-0.05	0.91
Dbf4	LEDGF/p75	Psip1 KO	0.11	0.83
		Double KO	-0.06	0.90
Tox4	LEDGF/p75	Psip1 KO	0.07	0.90
		Double KO	0.07	0.89
Nova1	LEDGF/p75	Psip1 KO	1.53	0.004
		Double KO	1.81	0.0006
Mcm7	LEDGF/p75	Psip1 KO	0.10	0.84
		Double KO	0.12	0.81
C3orf59	LEDGF/p75	Psip1 KO	-0.09	0.86
		Double KO	0.05	0.93
Map1a	LEDGF/p75	Psip1 KO	-0.50	0.32
		Double KO	-0.30	0.56
Iws1	LEDGF/p75	Psip1 KO	0.20	0.70
		Double KO	0.20	0.67
Sfrs1	LEDGF/p52	Psip1 KO	0.23	0.64
		Double KO	0.20	0.69

KO, knockout
