# Supplementary material for: Embryonic Lethality Due to Arrested Cardiac Development in Psip1/Hdgfrp2 Double-Deficient Mice
Source: PLoS One. 2015 Sep 14;10(9):e0137797. doi: 10.1371/journal.pone.0137797 (PMC4569352; doi:10.1371/journal.pone.0137797)
Supplement: S6 Table — (PDF) [file pone.0137797.s011.pdf]

S6 Table. Differential expression of *Hox* genes in pairwise tissue comparisons.

Gene	*Psip1* KO vs. ++/+g log_2 fold change	*P* value	Double KO vs. ++/+g (log_2 fold change)	*P* value
Hoxa1	n.d.	n.a.	n.d.	n.a.
Hoxa2	0.72	0.25	0.32	0.62
Hoxa3	0.57	0.30	0.63	0.26
Hoxa4	0.38	0.50	0.39	0.50
Hoxa5	0.84	0.10	0.52	0.32
Hoxa6	n.d.	n.a.	0.12	0.45
Hoxa7	0.90	0.08	0.99	0.06
Hoxa9	1.15	0.06	1.09	0.04
Hoxa10	0.80	0.10	0.79	0.12
Hoxa11	1.40	0.06	1.40	0.07
Hoxa13	0.19	0.70	0.35	0.50
Hoxb3	4.08	2.2×10^{-9}	3.04	9.0×10^{-6}
Hoxb4	1.88	6.9×10^{-4}	0.23	0.65
Hoxb5	2.65	8.3×10^{-6}	2.07	4.8×10^{-4}
Hoxb6	2.86	4.1×10^{-7}	2.18	8.5×10^{-5}
Hoxb7	2.27	4.6×10^{-5}	2.71	1.4×10^{-6}
Hoxb8	3.60	8.1×10^{-10}	2.75	1.6×10^{-6}
Hoxb9	3.07	2.1×10^{-8}	2.34	1.1×10^{-5}
Hoxb13	8.29	9.1×10^{-13}	8.55	9.7×10^{-14}
Hoxc6	2.37	1.4×10^{-5}	0.29	0.57
Hoxc8	2.46	7.2×10^{-6}	1.49	0.53
Hoxc9	4.37	4.9×10^{-13}	2.83	8.9×10^{-7}
Hoxd1	n.d.	n.a.	n.d.	n.a.
Hoxd3	n.d.	n.a.	n.d.	n.a.
Hoxd4	n.d.	n.a.	n.d.	n.a.
Hoxd8	1.29	0.012	0.83	0.10
Hoxd9	1.48	0.005	1.50	0.004
Hoxd10	1.30	0.01	1.60	0.002
Hoxd11	1.40	0.005	1.60	0.002
Hoxd12	2.60	5.0×10^{-5}	2.26	2.6×10^{-5}
Hoxd13	0.86	0.08	1.32	0.09

KO, knockout; n.d., not detected; n.a., not applicable
