# Supplementary material for: Embryonic Lethality Due to Arrested Cardiac Development in Psip1/Hdgfrp2 Double-Deficient Mice
Source: PLoS One. 2015 Sep 14;10(9):e0137797. doi: 10.1371/journal.pone.0137797 (PMC4569352; doi:10.1371/journal.pone.0137797)
Supplement: S7 Table — (PDF) [file pone.0137797.s012.pdf]

S7 Table. Alternatively spliced genes in double knockout versus control ++/+g samples.

Transcript	Associated phenotype
Tpm1	MXE
H2afy	MXE
Fn1	SE
Sptan1	SE
Rbm4	SE

MXE, mutually exclusive exon; SE, skipped exon
