# Supplementary material for: Embryonic Lethality Due to Arrested Cardiac Development in Psip1/Hdgfrp2 Double-Deficient Mice
Source: PLoS One. 2015 Sep 14;10(9):e0137797. doi: 10.1371/journal.pone.0137797 (PMC4569352; doi:10.1371/journal.pone.0137797)
Supplement: S1 Fig — (A) Genomic DNA from tails of mouse embryos were subjected to PCR using the indicated primer pairs. Primers AE2331 and AE2802 detect a 803 bp product from wild-type Psip1 DNA whereas exon 3 deletion yields a 324 bp fragment [15]. The 535 bp Hdgfrp2 product (primers AE2511, AE2512) is disrupted by gene trap insertion; primers AE3747 and AE3748, specific for the pGT2lfx vector, detect a 433 bp product in all cell types [10]. The migration positions of expected DNA products in bp and of mass standards in kb are shown to the left and right sides of the gels, respectively. DNA was detected by ethidium bromide staining. (B) Phenotypic characterization of Hdgfrp2 mRNA expression levels using the indicated primer pairs. P values from the indicated comparisons of expression levels are shown. (C) Psip1 mRNA expression levels. The data in panels B and C are averages and standard deviation of three independent experiments, with qRT-PCR samples conducted in duplicate for each experiment. (PDF) [file pone.0137797.s001.pdf]

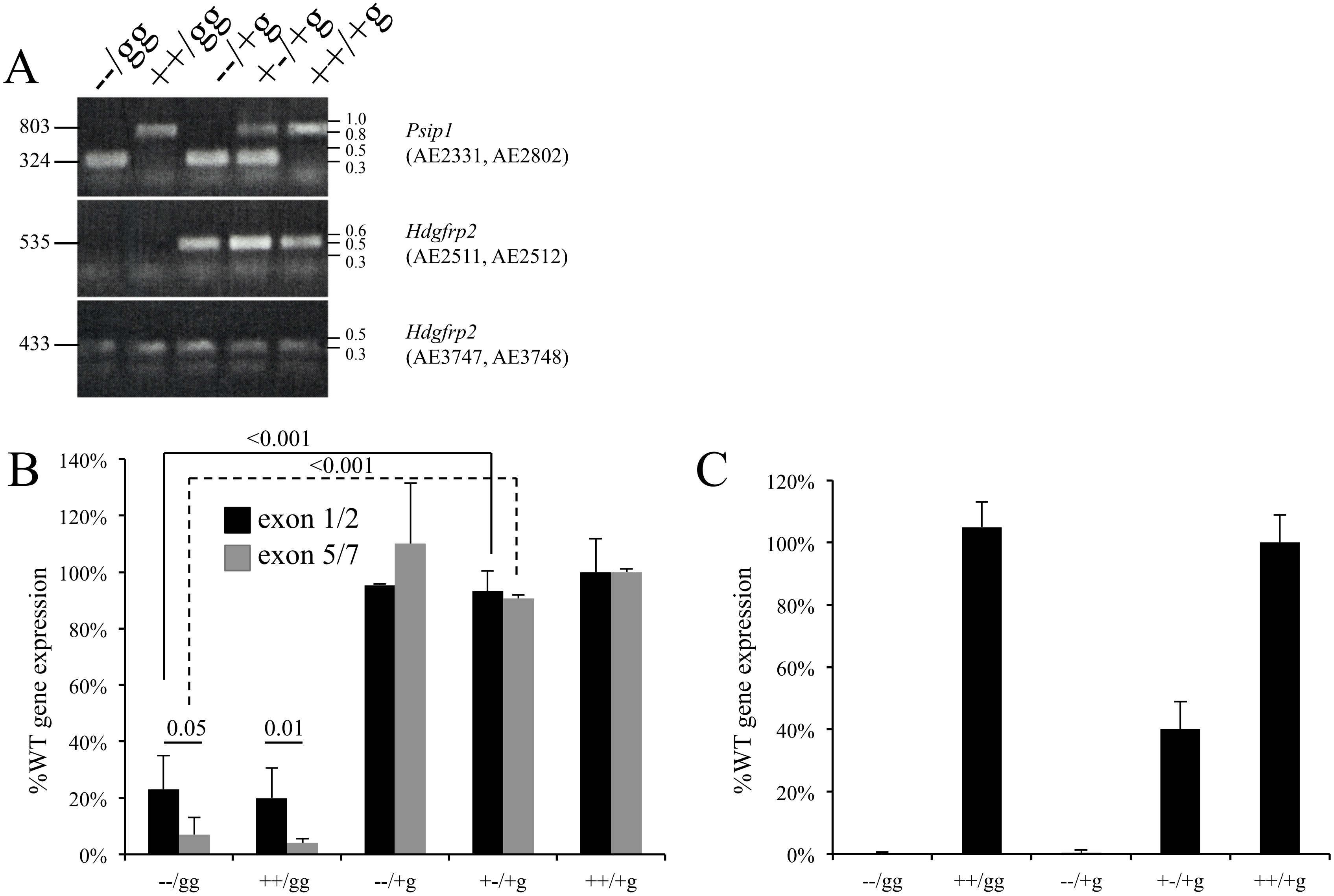
A
--/gg
++/gg
--/+g
+-/+g
++/+g
803
324
1.0
0.8
0.5
0.3
Psip1
(AE2331, AE2802)
535
0.6
0.5
0.3
Hdgfrp2
(AE2511, AE2512)
433
0.5
0.3
Hdgfrp2
(AE3747, AE3748)
B
<0.001
<0.001
exon 1/2
exon 5/7
0.05
0.01
%WT gene expression
140%
120%
100%
80%
60%
40%
20%
0%
--/gg
++/gg
--/+g
+-/+g
++/+g
C
120%
100%
80%
60%
40%
20%
0%
%WT gene expression
--/gg
++/gg
--/+g
+-/+g
++/+g

S1 Figure
